# Sex Specific Associations of BMI and Brain Regional Tau Deposition in a community‐dwelling cognitively normal older adult sample

**DOI:** 10.1002/alz.092970

**Published:** 2025-01-09

**Authors:** Joanna Dominguez, Oliver Cesar, Joshua L Gills, Anamarie Sogade, Laurel Y Browne, Elizabeth Weisgerber, Julian McBride, Elena Valkanova, Alfred Mbah, Luisa F Figueredo, Girardin A Jean‐Louis, Arjun V. Masurkar, Ricardo S. Osorio, Omonigho Michael Bubu

**Affiliations:** ^1^ NYU Grossman School of Medicine, New York, NY USA; ^2^ University of South Florida, Tampa, FL USA

## Abstract

**Background:**

Increased body mass index (BMI) in mid‐life is associated with a higher risk of Alzheimer’s disease (AD). Neurofibrillary tangles (NFTs), comprised of hyperphosphorylated aggregated tau protein, are the pathological hallmark of neurodegenerative tauopathies including Alzheimer’s Disease (AD). The relationship between sex‐dependent adiposity in late adulthood and tau NFT pathology remain inconclusive. We examined sex differences between BMI and tau‐PET pathology among cognitively normal individuals.

**Method:**

This cross‐sectional analysis consisted of 104 community‐dwelling cognitively normal older adults (mean±SD; males (*n* = 42): age = 72.8±6.5y; females (*n* = 62): age = 72.6±6.8y) from ongoing NYU longitudinal studies on memory and aging. Subjects completed clinical assessments and two PET scans (FBB and ^18^F‐ PI2620). Standard uptake value ratio (SUVR) values from images were computed using FreeSurfer, and normalizing to cerebellar grey matter. For tau, data were analyzed from composite regions corresponding to Braak pathological stages I–VI, (Braak I: entorhinal cortex; Braak II: hippocampus; Braak III: parahippocampal gyrus, amygdala, fusiform gyrus, lingual gyrus; Braak IV: neocortical regions including the insular cortex, medial temporal gyrus; Braak V: superior temporal gyrus; Braak VI: occipital neocortex), and control brain regions not expected to contain NFTs including the pericalcarine cortex, corpus callosum, and caudate nucleus. Linear mixed effects regression models examined association between BMI, sex, BMI*sex, PET SUVR amyloid & tau. Models were adjusted for age, race, amyloid and ApoE status.

**Result:**

Relative to females, males were on average slightly more educated (males, females; 17.0±2.4y, 16.6±2.1y), had similar BMI (29.6±10.3 kg/m^2^, 29.5±10.8 kg/m^2^), and had higher blood pressure (Systolic/diastolic; 168.4/116.3 mmHg, 156.9/100.7 mmHg). Sex and BMI were each associated with SUVR tau and the BMI*sex interaction was significant (*p*<0.05 for all). Males with higher BMI, showed increased SUVR tau burden (β[IV] = β[SE}; β[BMI*sex] = 0.0054[0.0025], *p* = 0.031), corresponding to Braak stage I (β[BMI*sex] = 0.0122[0.0055], *p* = 0.032), Braak stage III (β[BMI*sex] = 0.0054[0.0025], *p* = 0.034), Braak stage IV (β[BMI*sex] = 0.0058[0.0020], *p* = 0.006), Braak stage V (β[BMI*sex] = 0.0048[0.0019], *p* = 0.014), and Braak stage VI (β[BMI*sex] = 0.0039[0.0017], *p* = 0.026).

**Conclusion:**

In this sample of community‐dwelling cognitively normal older adults, higher BMI in males was associated with increased tau NFT burden. Thus, excessive adiposity in late‐life may contribute to Alzheimer’s disease pathology for males.

